# Oncologist approaches to communicating uncertain disease status in pediatric cancer: a qualitative study

**DOI:** 10.1186/s12885-022-10190-6

**Published:** 2022-10-31

**Authors:** Amy S. Porter, Cameka Woods, Melanie Stall, Srilakshmi Velrajan, Justin N. Baker, Jennifer W. Mack, Erica C. Kaye

**Affiliations:** 1 St. Jude Children’s Research Hospital, Memphis, TN USA; 2 The University of Texas Southwestern Medical Center, Dallas, TX USA; 3 The University of Memphis, Memphis, TN USA; 4 Dana-Farber Cancer Institute, Boston, MA USA; 5 Boston Children’s Hospital, Boston, MA USA; 6 Division of Quality of Life and Palliative Care, Department of Oncology, St. Jude Children’s Research Hospital, 262 Danny Thomas Place, Mail Stop 1121, 38105 Memphis, TN USA

**Keywords:** Pediatrics, Communication, Prognosis, Uncertainty

## Abstract

**Background:**

Most patients with cancer and their caregivers desire honest, clear prognostic communication, yet oncologists often disclose prognosis inconsistently. Prognostic communication becomes even more challenging when disease progression is unclear or equivocal. Presently, oncologist approaches for discussing uncertain disease findings are poorly understood.

**Methods:**

In this prospective, longitudinal study, we audio-recorded serial disease reevaluation conversations between children with high-risk cancer, their families, and their primary oncologists over 24 months and conducted content analysis at recorded timepoints when oncologists categorized disease progression as equivocal.

**Results:**

Of the 265 medical discussions recorded across the illness course for 33 patient-parent dyads, a total of 40 recorded discussions took place at equivocal timepoints, comprising > 500 min of medical dialogue. Prognosis talk encompassed < 3% of dialogue and was absent in nearly half of equivocal discussions (17/40, 42.5%). Curability statements were identified in only two conversations. Inductive content analysis of dialogue revealed four distinct patterns for communicating equivocal disease status: (1) up-front reassurance, (2) softening the message, (3) describing possible disease progression without interpretation, (4) expressing uncertainty without discussing the bigger picture.

**Conclusion:**

Oncologists rarely discuss prognosis with children with high-risk cancer and their families at timepoints when disease progression is not definitive. Formal guidance is needed to better support oncologists in navigating uncertainty while sharing honest, person- and family-centered information about prognosis.

**Supplementary information:**

The online version contains supplementary material available at 10.1186/s12885-022-10190-6.

## Background

Most patients with cancer and their families want to receive honest communication about prognosis from their medical team, including truthful disclosure about poor prognosis. [1–3] Sharing prognostic information, however, is rarely straightforward, and evidence suggests that oncologists struggle to discuss prognosis directly, often veiling prognostic information in vague language or avoiding prognostic disclosure altogether. [4–6] Individual preferences and cultural differences also influence the ways that patients, families, or clinicians wish for prognostic information to be shared, [7–12] adding further complexity to already challenging terrain.

Navigating communication about prognosis becomes even more difficult in the setting of uncertainty. Medical professionals often struggle to discuss prognosis directly when the outcome is not definite. [13–15] In analyses of physician-patient encounters, medical oncologists rarely discussed prognostic uncertainty. [15–17] When simply reviewing hypothetical patient vignettes, most oncologists felt comfortable telling the patient about an incurable disease, yet fewer were willing to disclose uncertainty regarding life expectancy. [18].

Over the past decade, however, dexterity in navigating prognostic uncertainty has become increasingly integral to provision of cancer care. In spite, or perhaps because, of increasingly sophisticated diagnostics and therapeutics, uncertainty with anticipating outcomes for patients with high-risk cancer is common. [19] In pediatric oncology, in particular, predicting outcomes for children with rare cancers treated with novel therapeutics is challenging, [20, 21] and communication approaches for navigating this uncertain space remain poorly understood.

The U-CHAT (Understanding Communication in Healthcare to Achieve Trust) trial was designed to identify and describe strategies used by pediatric oncologists to communicate prognostic information with patients and families across advancing illness. In this analysis, we focused on disease reevaluation conversations between pediatric oncologists, patients with high-risk cancer, and their parents, which oncologists categorized as “equivocal,” meaning data were ambiguous and difficult for the oncologist to characterize as either “good news” or “bad news.” Through this targeted analysis, we aimed to (1) quantify the frequency of prognostic communication in the setting of equivocal disease status for children with high-risk cancer and (2) identify thematic patterns in oncologist approaches for navigating prognostic information when disease progression is ambiguous.

## Methods

An interdisciplinary team of pediatric oncology and hospice and palliative medicine experts collaborated with the St. Jude Children’s Hospice Bereaved Parent Steering Council to develop the U-CHAT trial. The protocol was approved by the St. Jude Children’s Hospital Institutional Review Board (U-CHAT [Pro00006473]; approval date: July 12, 2016. Data were collected between 2016 and 2020. We present study methods and findings following the Consolidated Criteria for Reporting Qualitative Research (COREQ) reporting guideline and checklist (Supplemental Table 1). [22] Data included in this analysis included recorded disease reevaluation conversations between oncologists and patients’ parents, as well as surveys and recorded interviews of oncologists following those conversations.

Participant Enrollment and Data Collection.

Detailed eligibility criteria, enrollment, and informed consent processes were published previously [6, 23–25] and are summarized in Table 1. Briefly, patients with high-risk solid tumor cancers and their families were identified by the research team and approached if their primary oncologist estimated survival as ≤ 50% and expected the patient to have ≥ 2 future disease reevaluation timepoints. Following a standardized informed consent process, patient-parent dyads were enrolled on study and followed longitudinally for 24 months from last disease progression or until death, whichever occurred first. All medical discussions where the oncologist planned to disclose findings from disease reevaluation studies (e.g., laboratory tests, imaging, pathology, etc.) were audio-recorded serially. Conversations were recorded in the clinic or hospital setting, as well as rarely via telephone if patients/families were unable to come to the hospital to discuss disease reevaluation findings. Following each discussion, the recorded conversation was categorized by the primary oncologist as “good news” (i.e., no evidence of disease progression), “bad news” (i.e., clear evidence of disease progression), or “equivocal news” (i.e., ambiguous, unclear findings; unable to definitively describe as good or bad news).

**Table 1:** Eligibility criteria, recruitment, and informed consent processes

**Eligibility Criteria**
• Eligible healthcare professionals: Primary oncologists who provided medical care to solid tumor patients at the study site. Other eligible providers: Non-primary oncologist healthcare professionals (e.g., fellows, students, nurses, psychosocial providers) who attended a recorded disease reevaluation conversation for enrolled study patients (participation limited to attendance during recording). • Eligible patients, parents, and others: Aged 0–30 years, “high-risk” solid tumor cancer diagnosis, with primary oncologist estimating survival at ≤ 50% and projecting ≥ 2 future disease reevaluation timepoints. Legal caregiver of eligible patient, aged ≥ 18 years, English language proficiency, planned to accompany patient to medical visits. Family or friends of an enrolled patient-parent dyad who attended a recorded disease reevaluation conversation (participation limited to attendance during recording).
**Recruitment & Informed Consent**
• Healthcare professionals: The Principal Investigator (PI) sent emails to a convenience sample of all eligible primary oncologists at the study site to introduce the study and determine interest in participating; the PI then met one-on-one or in small groups with eligible oncologists to describe the study and complete the informed consent process. Eligible non-primary oncologist healthcare professionals were introduced to the study by the PI or research team member during clinic or office time preceding a scheduled recording, and informed consent was obtained. • Patients, families, and friends: Eligible patient/parent dyads were identified by the research team through review of outpatient clinic schedules and institutional trial lists and confirmed by the primary oncologist. Patient-parent dyads were approached by a member of the research team during a clinic visit that was unrelated to disease reevaluation timepoint to determine interest in participation. If interested, the study was described in detail. Dyadic enrollment necessitated agreement from both patient and parent. Patients aged ≥ 12 years provided assent, and patients aged ≥ 18 years and parents provided consent. Eligible family/friends were introduced to the study by the PI or research team member prior to recording the visit, and verbal consent was obtained.

In addition to collecting recorded dialogue, following any “bad news” disease reevaluation discussions, oncologists and parents participated in surveys and audio-recorded semi-structured interviews conducted by research team members trained in qualitative interviewing (CW, EK), using prompts read-aloud to participants that had been pilot tested previously. Interview duration averaged 20 min (range 5 min to > 2 h, dictated by participant preference). Both surveys and interviews included a validated question previously tested in this population: “How likely do you think it is that your child [or patient] will be cured of cancer?” [26–29] Data about patient demographics and illness course were abstracted from the electronic medical record using a standardized tool, and interviewers wrote memos following interviews.

Codebook Development, Coding, Adjudication, and Analysis.

A team of pediatric oncology and palliative medicine clinicians and researchers (Supplemental Table 2) reviewed the literature and found limited frameworks to conceptualize prognostic communication in pediatric cancer. Building upon adult oncology communication standards, [30, 31] the American Society of Clinical Oncology’s communication consensus guidelines, [32] and the Prognostic and Treatment choices scale, [33] the team developed an *a priori* codebook to explore prognostic communication between oncologists, children with high-risk cancer, and their families across evolving illness. The codebook categorized prognostic communication into six language domains: prognostic uncertainty, assessing prognostic understanding, disease changing for the worse, best- and worst-case scenarios, survival time, and curability. Codes and definitions are presented in Supplemental Table 3.

To ensure consistency in code application, qualitative analysts (CW, MS, SV, EK) independently pilot-tested the codebook across a series of medical dialogue recordings until consensus was reached. The research team (CW, MS, SV, JB, EK) met to reconcile variances and achieve consensus, modifying the codebook as needed to improve dependability, confirmability, and credibility of independent codes. [34] The codebook was finalized following deep review of sufficient raw data to reach saturation, with no new concepts emerging from transcripts. [35].

Content analysis was conducted per COREQ guidelines, [22] using MAXQDA to organize data (VERBI Software, 2020). [36] Coding was performed by four analysts with training in and experience with content analysis (AP, CW, MS, SV), with each recording coded by at least two independent coders. The research team held weekly meetings for review of coding variances and third-party adjudication to reach consensus. Consistency in code segmentation also was reviewed to ensure a standardized approach.

To maximize opportunities for examination of prognostic communication in the context of uncertainty, this analysis focused on recorded disease reevaluation discussions categorized by oncologists as “equivocal.” Across equivocal discussions, code frequency, temporal duration, and distribution were analyzed and reported as descriptive statistics (AP, CW, EK). Iterative review and serial memo writing of coded dialogue [37] (AP, EK) informed the development of inductive themes describing the communication approaches used by oncologists to navigate discussion about unclear disease status.

## Results

A total of 265 medical discussions were recorded across the illness course for 33 patient-parent dyads, comprising more than 4,000 min of recorded dialogue. Data on patient-parent dyads who declined enrollment in U-CHAT were previously published; [6, 25] briefly, 17% of approached dyads (n = 7 dyads) did not enroll due to hesitation or refusal by the patient (n = 3), parent (n = 3), or both (n = 1). Refusal rates did not appear to disproportionately exclude dyads based on race or ethnicity, [6, 25] although small numbers precluded formal scrutiny.

More than half of participating dyads experienced one or more equivocal disease reevaluation timepoints during the study period (17/33, 51.5%); of these, about half (9/17) had more than one equivocal discussion (mean 3.6 equivocal discussions per dyad, range 2–9). Approximately 15% of recorded conversations (40/265) and 12.5% of total dialogue time (510/4,050 min) took place at timepoints with equivocal disease reevaluation findings, comprising > 500 min of medical dialogue and making 40 the denominator for this analysis. All participating oncologists (n = 6) presented equivocal findings to patients and families in at least one disease reevaluation discussion.

Of the dyads involved in equivocal discussions, most were white (15/17, 88.2%), and gender was roughly equivalently divided. Adolescents and young adults (aged ≥ 12 years) comprised more than half of patient participants (9/17, 52.9%). Full participant demographic variables are presented in Table 2. No participants formally dropped out of the study, although one dyad transferred care to another institution prior to death. Most equivocal discussions (34/40, 85%) were followed by disease progression within the 24-month study duration. Among the 17 dyads who experienced at least one equivocal discussion, 13 patients had disease progression, and at the time of publication of this paper, all 13 had died.

**Table 2:** Demographic characteristics for participants with recorded equivocal discussions

Variable	n (%)
**Patient (n=17)**	
Gender	
Female	8 (47.1)
Male	9 (52.9)
Race	
White	15(88.2)
Black	2 (11.8)
Ethnicity	
Hispanic	0 (0)
Non-Hispanic	17(100)
Age at Diagnosis	
0-2 years	2 (11.8)
3-11 years	6 (35.3)
12-18 years	8 (47.1)
19+ years	1 (5.9)
**Parent (n=17)**	
Gender	
Female	15(88.2)
Male	2 (11.8)
Role	
Mother	14(82.4)
Grandmother	1 (5.9)
Father	2 (11.8)
**Pediatric Oncologist (n=6)**	
Gender	
Female	3 (50)
Male	3 (50)
Race	
White	6 (100)
Black	0 (0)
Ethnicity	
Hispanic	0 (0)
Non-Hispanic	6 (100)
Years in Clinical Practice	
1-4 years	2 (33)
5-9 years	2 (33)
10-19 years	0
20+ years	2 (33)

Frequency of prognostic communication in equivocal disease reevaluation discussions.

Frequencies and time duration of dialogue coded as prognostic communication (prognostic uncertainty, assessing prognostic understanding, disease changing for the worse, best- and worst-case scenarios, survival time, curability) are presented in Table 3, with representative quotes for each code presented in Table 4. Prognostic communication codes were applied 80 times across 40 equivocal discussions (median 1 code per recorded conversation, range 0–13), totaling < 14 min of prognosis discussion over 510 min of total dialogue time, or 2.9% of total minutes of recorded conversation. Given that this analysis targeted equivocal discussions, it was unsurprising that the most dominant code identified was “prognostic uncertainty” (Table 5). Although oncologists labeled conversations as “equivocal news” rather than “bad news,” the code for “disease changing for the worse” was the code that constituted the most recorded dialogue time across all recordings. Most dialogue coded as “disease changing for the worse” described specific disease reevaluation findings consistent with minimal disease progression within a “big picture” setting that was described as unclear or equivocal.

**Table 3:** Descriptive statistics for prognostic communication codes in equivocal discussions

Code Name	Code Frequency			Code Time			
	No. of codes across all equivocal recordings	Median (range) of codes per recording	No. (%) recordings including at least 1 code	Total time of coded dialogue across all recordings	Median (range) of time coded per recording	% of coded time (time for each code per total prognostic communication time)	**Coverage** (time for each code per total dialogue time)
Prognostic uncertainty	35	0 (0–6)	19/40 (47.5%)	6 min 23 s	0 Sect. (0 s-2 min 2 s)	~ 46%	**1.3%**
Disease changing for the worse	35	0 (0–5)	17/40 (42.5%)	5 min 48 s	0 Sects. (0–47 s)	~ 42%	**1.1%**
Best- and worst-case scenarios	4	0 (0–1)	4/40 (10%)	54 s	0 Sects. (0-18.5 s)	~ 6%	**0.2%**
Assessing prognostic understanding	4	0 (0–3)	2/40 (5%)	16 s	0 Sects. (0-8.8 s)	~ 2%	**0.1%**
Curability	2	0 (0–1)	2/40 (5%)	46 s	0 Sects. (0-33.9 s)	~ 5%	**0.2%**
Survival time	0	0 (0)	0/40 (0%)	0 s	0 Sect. (0 s)	0%	**0%**
Total	80	1 (0–13)	23/40 (57.5%)	13 min 45 s	0 Sect. (0 s-2 min 36 s)	100.0%	**2.9%**

Total recorded time: 8 h, 29 min, 45 s

**Table 4:** Representative quotes for prognostic communication codes

Code	Example language coded
Prognostic uncertainty	• “The bone marrow looked a little bit different - but it didn’t really look different on PET scan, so I don’t know what to make of that at all.” • “These little things, I’m not even sure what they are. I’ll show you the pictures. Um, they definitely don’t light up at all, but they are so tiny and the radiologist doesn’t even know what to say about them either.” • “It looks [like] maybe a collection of fluid kind of along the spinal canal in that lower part, we aren’t entirely sure what that is, or why it’s there but it doesn’t really look like tumor either, so we are not entirely sure what to make of that other than we know that you’re doing well.” • “Some places that we worry that it might be getting worse - but nothing that I can say for sure.”
Disease changing for the worse	• “Remember this? Last time there was maybe this new little thing on the other side. That is there and maybe looks a teensy bit bigger. Okay? There are no other new spots in the lungs, and that being said, I’m talking like a millimeter or so bigger - but definitely a little bit bigger.” • “One of those areas has turned dark…which looks exactly like the original tumor when it came back, so that’s why I want to do a PET.”
Best- and worst-case scenarios	• “We can hope it’s an infection that obviously isn’t bothering her, but I’m very worried that it could the cancer.” • “Again, I wish I could walk in and say, hey everything disappeared, that would be the best news, so I don’t have that news, but the worse news would be that things are worse and that is definitely not the case
Curability	• Clear: “This is getting better. Is this medicine going to cure her? The answer is very likely not. We know that. But it’s giving her very, very good quality of life, with relatively little interruptions.” • Cloudy: “Our first worry is God forbid this is awful thing comes back, and if it comes back this early we’re in big trouble. You know after all the treatment he’s had, you know.”
Assessing prognostic understanding	• “Ask me more questions because you don’t sound satisfied. You just said ‘ok,’ but you need to talk to me a little more.” • “Does that make sense? Are we sure?”
Survival time	*No codes*

**Table 5:** Patterns of prognostic communication in equivocal discussions

Pattern	Characterization	Example
*Up-front reassurance*	Opening the conversation and/or repeatedly stating that the patient is doing well or okay despite equivocal results	• “We have good news.” • “I don’t think [this is disease]. Very likely, it is not.”
*Softening the message*	Use of modifiers to soften the message about possible disease progression	• “Let me tell you what I found, I don’t want you to start freaking out…everything looks pretty stable on the PET scan, ok there is a very, very, very, tiny, small area on the left femur and a very small area on the right knee, in retrospect I think they were there before, so I am not very worried about them.” • “It’s not changing by leaps and bounds; it’s changing very slowly over time. It’s gotten just a little incrementally slightly bigger since the last time we looked at it.”
*Describing possible disease progression without interpretation*	Detailed description of disease reevaluation findings (i.e., imaging) without connection to prognosis	• Worsened imaging: “The stuff in her lungs is worse.” • Stable/improved imaging: “Chest looks great. You still have on the one side that nodule; it is definitely not bigger, so that is good. And there are no new spots anywhere in your chest.” • Uncertain change in imaging: “I mean there’s one little spot that he had when he came in around the second rib. That we’ve been watching, and that’s getting better every time. The rest of it in the whole area [on] the MRI shows these abnormalities that could be tumor if you just look at that in isolation.”
*Expressing uncertainty without context*	Direct statements of uncertainty without statements of concern about disease progression	• “The bone marrow looked a little bit different [on MRI], but it didn’t really look different on PET scan, so I don’t know what to make of that at all.” • “[In] some places we worry that it might be getting worse - but nothing that I can say for sure.”

Prognostic communication dialogue was present in just over half of recorded equivocal discussions (23/40, 57.5%), and when codes were analyzed individually, each code was found in < 50% of recordings: “prognostic uncertainty” 47.5% (19/40), “disease changing for the worse” 42.5% (17/40), and “assessing prognostic understanding” 5% (2/40). Fewer than 10% of recorded equivocal conversations included dialogue addressing whether the cancer could be cured: “best- and worst-case scenarios” was identified in 10% of conversations (4/40), “curability” in 5% (2/40), and no discussions included “survival time” codes. Across all equivocal discussions, the “curability” code was applied a total of twice and the “assessing prognostic understanding” applied a total of four times. When the latter code was applied, the depth and focus with which prognostic understanding was explored was limited (Table 4), representing a cursory assessment of patients’ and families’ awareness of prognosis.

Oncologist communication patterns in settings of uncertainty.

Inductive content analysis of prognostic communication dialogue revealed four thematic patterns for how oncologists shared prognostic information when disease reevaluation findings were worrisome yet lacked evidence of frank disease progression (Table 5).

Up-front reassurance: Although oncologists categorized these discussions as “equivocal” to the research team, when talking with patients and families, they often led with reassurance about the uncertain findings. For example, oncologists frequently opened conversations with a positive statement to offer relief for the waiting family:

“So, everything looks stable on scans, okay. I don’t have the bone marrow test back, but his [labs] are normal. So, I think everything’s where we were a month ago in terms of scans.”

One oncologist opened the conversation with “good” news despite privately classifying the findings as “equivocal”: “So, I know you just want to hear about scans, so we are going to start talking about that first. Everything is stable, and there is nothing new. So, that’s good.” That oncologist went on to relativize the positive framing as good but not “the best”:

I wish that I could say – I mean, the best thing would be if I came in and said everything is gone. So, I don’t want to pretend like that wouldn’t be the best news – that would be the best news.

Softening the message: While conveying equivocal findings, oncologists softened the message of possible disease progression by using minimizing modifiers to downgrade worry. For example, one oncologist said:

The CT of the chest shows a very, very small little nodule which is about 2 mm on the left lung. That maybe just a little blood vessel within the lungs…so what we need to do is just follow that.

Oncologists also used emphatic language (“they definitely don’t” and “so tiny”) to minimize the weight of uncertain data:

These little things, I’m not even sure what they are. I’ll show you the pictures – they definitely don’t light up at all, but they are *so tiny*, and the radiologist doesn’t even know what to say about them either.

Describing possible disease progression without interpretation: Many oncologists described disease reevaluation findings (e.g., laboratory tests, imaging, pathology, etc.) in detail but did not interpret how the findings may impact prognosis and curability. For example, oncologists pointed out new lesions (“So there is one little spot in your clavicle, which is a fancy word for your collar bone, that is bright…”) or increases in lesion size (“The one over here is a little bit more elongated than it was before but not by a huge extent”) often without connecting these findings to the bigger picture or explaining what the lesions could mean for the patient’s future life.

Expressing uncertainty without discussing the bigger picture: Oncologists offered statements of uncertainty without expressing concerns about the possibility of disease progression or anchoring the moment of uncertainty in the context of a prior high-risk diagnosis. In this approach, language like “I just don’t know” or “I just can’t know” were often used. At times, oncologists expressed their hesitation frankly: “I certainly don’t feel 100% confident, like, I don’t want to say this is [disease] because I don’t know that.” Similarly, another oncologist used the phrase “not entirely sure” repeatedly in interpreting findings:

It looks maybe a collection of fluid…We *aren’t entirely sure* what that is or why it’s there, but it doesn’t really look like tumor either, so we are *not entirely sure* what to make of that other than we know that you’re doing well.

Co-occurrence of patterns: The “describing possible disease progression without interpretation” pattern frequently occurred concurrently with “softening the message” or “expressing uncertainty without discussing the bigger picture” patterns. Specifically, when oncologists focused on describing findings in detail, they used modifiers to minimize concern or emphasized inability to confirm bad news:

That’s the one we have been following, and when we look at that one…the difference is a couple of millimeters. Um, so it’s not - I can’t say that it has decreased in size, but it has not gotten bigger to a degree that I could say that this is clearly, you know, something that is blowing up and progressing.

While oncologists rarely voiced concerns about disease progression during recorded equivocal discussions, data from surveys and interviews showed that oncologists generally believed that their patients’ disease would progress and likely be incurable for most patients. Specifically, all 6 participating oncologists completed surveys and interviews following disease progression for all 13 patients who progressed while on study; for each of these patients, the oncologist estimated odds of cure to be very low or zero. In response the question: “How likely do you think it is that your patient/child will be cured of cancer?,” oncologists offered a range of similar responses: “Nearly impossible, but we can hope;” “I do not think she will be cured unfortunately;” “I would still say less than 10%, but we would always love to be proven wrong;” and “I do not think she’ll be cured…less than 5%.” One oncologist explored the complexity of interpreting disease reevaluation data and the challenge of sampling error when responding to this question:

Zero, nothing. We barely got her to transplant…She never cleared her marrow, and the last marrow, by a miracle, it came back negative. I think it was just sampling error. I think there was always disease there.

Another oncologist alluded to the inevitability of disease spread even without visible evidence on imaging:

I think it’s unlikely he’ll have long-term cure. I think he might have a period of disease- free, as best we can tell in terms of pictures. Obviously, you know, if he has disease in his lungs, he probably has micro-mets that we can’t see…

## Discussion

Pediatric oncologists often face prognostic uncertainty, particularly when interpreting indefinite or equivocal findings. In this qualitative study, equivocal conversations occurred relatively often: more than half of patient-parent dyads experienced one or more equivocal conversations, and all oncologists participated in discussions about equivocal findings. The prevalence of this experience suggests the need for oncologists to receive training and be prepared to navigate communication about disease status in the setting of uncertainty.

Notably, all patients in this study were considered “high-risk,” with their primary oncologist estimating survival at ≤ 50% to qualify patients for enrollment. Despite patients’ high-risk status, little discussion of prognosis occurred during equivocal timepoints where disease progression was suspected but not definitive. These findings corroborate prior exploratory work suggesting that opportunities exist for oncologists to consider “seed planting” communication approaches, including anticipatory discussion to explore a patient’s or family’s hopes, worries, and goals with the intention of laying groundwork for future conversations about prognosis. [6].

In lieu of seed planting, we found that oncologists are more likely to reassure, soften the message, focus on disease or treatment details without prognostic interpretation, or express uncertainty without referencing the “big picture” context during discussions about equivocal disease status, even in the setting of anticipated poor prognosis. This phenomenon of “kicking the proverbial can down the road” likely has multifactorial origins. For example, oncologists historically self-report fears that discussing uncertainty may harm therapeutic alliance or steal hope from patients and families. [38–43] Contrary to this assumption, however, patients and parents who received honest information about poor prognosis were more likely to report feeling peace of mind, trust in the physician, and hope, [26, 27, 44–46] suggesting that some of these fears may be unfounded. At the same time, the impact of uncertainty on patients’ and parents’ experiences of prognostic communication remains understudied.

Oncologists’ personal values and attributes also may influence their communication approaches. An oncologist’s own tolerance for uncertainty has been shown to be significantly associated with willingness to discuss an uncertain prognosis with patients and families. [18] Additionally, oncologists describe awareness of “collusion” as a common phenomenon where stakeholders avoid direct conversation about prognosis as part of an unspoken dance. [47] The premise of this phenomenon, however, rests upon an assumption that the patient and family share the same understanding of prognosis as the oncologist. Counter to this, previous studies demonstrate that concordance in prognostic understanding between oncologists and parents of children with advanced cancer is often poor. [6, 48, 49] Collusion, by definition, is only possible in settings in which both parties know and understand the prognosis.

Patients’ and families’ preferences for discussing prognosis in the setting of uncertainty and equivocal disease reevaluation data are not well understood, although preliminary work suggests that families recognize the challenges and benefits of having direct conversation about prognostic uncertainty. [13] Notably, most adolescents with cancer and parents of children with cancer want to hear direct, truthful, individualized, and regular communication about prognosis across the illness course [21, 44, 50–52] and seek support in applying population-level prognostic information to their child’s specific trajectory. [52] For personal or cultural reasons, some families prefer for prognostic information to be withheld from the patient; yet data suggest that, when asked directly, patients often express a preference for their physician to be honest, even as families strive to protect them from stressful information. [53].

**Fig. 1:**
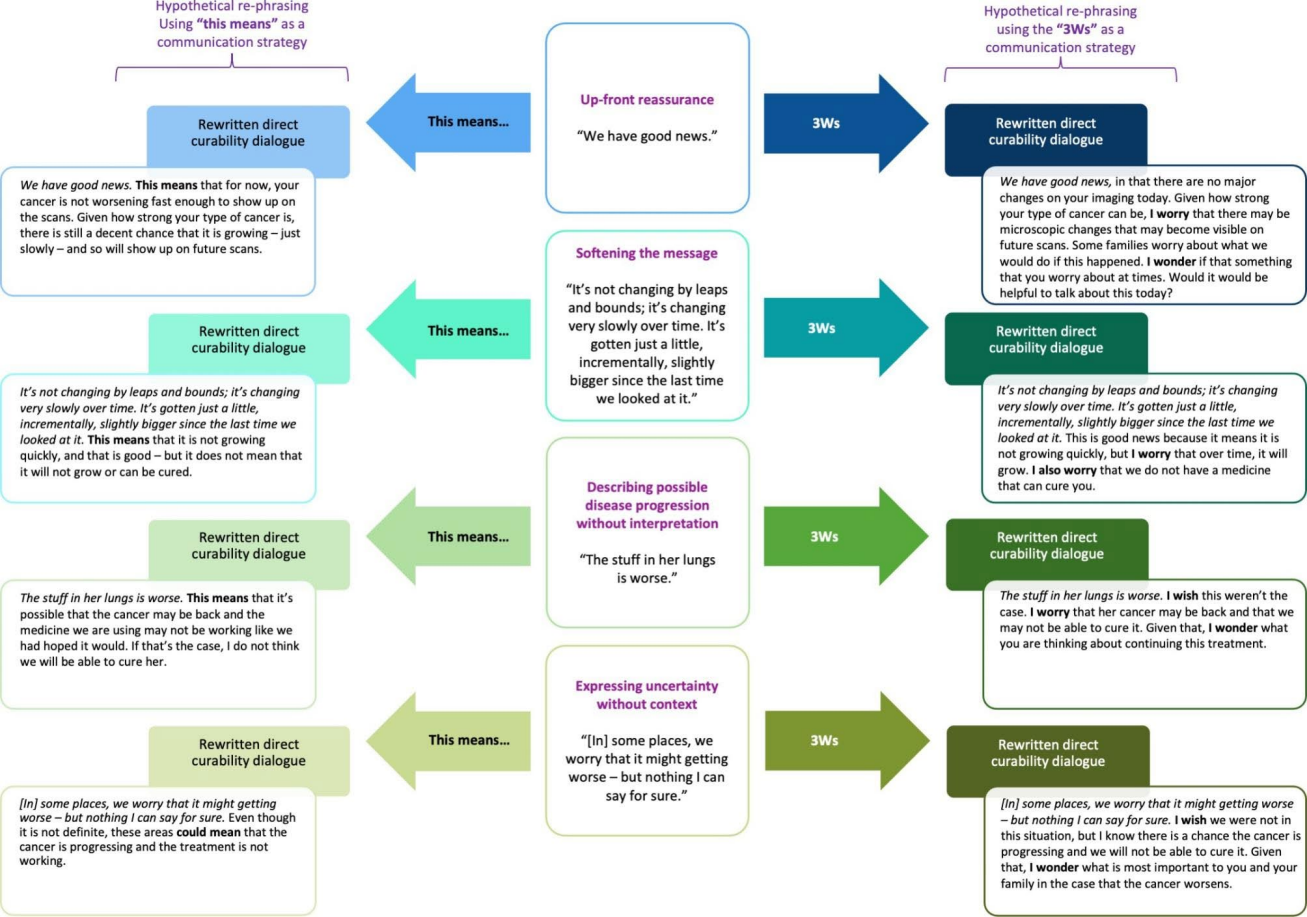
Applying communication strategies for prognostic communication in settings of equivocal news. Patterns of prognostic communication in disease reevaluation conversations conveying equivocal news are specified with recommendations for re-stating news using two communication strategies: (1) “this means” and (2) the “3Ws” (e.g., “I wish, I worry, I wonder”). Suggestions for alternative phrasings are based on the authors’ collective clinical experiences

Presently, few communication guidelines exist to support pediatric oncologists in disclosing uncertain or equivocal disease reevaluation findings. Resources and training should emphasize the value of person-centered communication, with awareness of the importance of empowering adolescent and young adult patients to participate in conversations and decision-making in alignment with their preferences and values. [54, 55] Two communication tools used frequently in palliative medicine practice and pedagogy may be useful in guiding conversations conveying equivocal information to patients of varying ages and their families: “this means” and the “3Ws.” In Fig. 1, we illustrate how the patterns of prognostic communication used by oncologists in this study might be reframed using “this means” and the “3Ws” (“I wish…”, “I worry…”, “I wonder…”) statements to help navigate communication during uncertain timepoints in the setting of an anticipated poor prognosis. In particular, “I worry” statements offer an effective strategy for “seed planting” [6] to help clinicians broach difficult prognostic communication in a gentle, step-wise approach across time.

We also recognize that meaningful gaps exist between what is known (and visible on disease surveillance imaging) and what is perceived, interpreted, or discerned based on the oncologist’s deep experience and knowledge. Oncologists may describe discussions as “equivocal” because the concrete evidence before them does not afford sufficient certainty to label the discussion as “bad news.” We advocate for increasing emphasis on communication training for oncologists to help them navigate the space between the visible and the invisible to communicate uncertainty with care and intentionality. Experiential learning [56] and role-play with standardized patients and/or bereaved parent educators [57] allows clinicians (e.g., oncologists, other physicians, and members of interprofessional teams, including social workers, psychologists, nurse practitioners, nurses, and others) to practice strategies for communicating about prognosis when disease progression is ambiguous, yet overall survival is unlikely.

Study findings should be interpreted in the context of limitations. Single-site design limits generalizability; however, qualitative research inherently does not aim for generalizability, and sample size was adequate for saturation of concepts. Sampling bias should be considered, as the study was conducted at a pediatric cancer center that recruits for phase I/II trials, and oncologist communication approaches could be influenced by a focus on cancer-directed treatments. Despite purposive sampling, racial and ethnic diversity was limited, which necessitates prioritization in future work. For example, Black patients comprised 11% of participants in this study, but they comprise 15% of the institution’s patient population and 16% of the state population. Similarly, purposive sampling was not successful in increasing Hispanic/Latino participation, due in part to eligibility criteria that precluded non-English speaking dyads. Similarly, lack of diversity in oncology faculty at the institution limited ability to represent different perspectives in oncology participants. Subsequent research protocols building upon these data have taken steps to proactively increase diversity across recruitment.

Rarely, discussions were not recorded due to logistical issues or at the request of the participating patient or parent; missing data could influence inductive analysis, although a few missing timepoints in the context of thousands of recorded minutes are less likely to influence data synthesis. Our analysis focused more on oncologist communication about prognosis, rather than on parent responses or questions; we underscore that patient/parent questions can change how an oncologist communicates about prognosis, and examination of patient/parent language to prompt or shape oncologist responses is an important consideration to inform future research. We did not conduct analyses stratified by patient age to explore potential variances in communication patterns influenced by age, and this query deserves future investigation. Finally, the phenomenon where oncologists defined a conversation as “equivocal” (instead of labeling it as “bad news”), yet dialogue frequently was coded as “disease changing for the worse,” is deserving of further attention. In this analysis, parsing out these nuances would have required extensive reading “between the lines,” as oncologists did not directly verbalize this discrepancy and researchers did not attempt to elicit or probe it in real time since the finding was identified during later targeted analyses. We advocate for purposeful exploration of this discrepancy in future work.

During conversations about equivocal disease reevaluation findings, pediatric oncologists rarely discussed prognosis directly with patients and families. Given that equivocal findings occurred frequently for pediatric patients with high-risk cancer, formal guidance is needed to better support oncologists in navigating uncertainty while sharing honest, person- and family-centered information about prognosis. Patient, parent, and oncologist perspectives and preferences should inform the design and evaluation of clinical communication tools to support prognostic communication across the illness course.

## Electronic supplementary material

Below is the link to the electronic supplementary material.

Supplementary Material 1

## Data Availability

Deidentified selections of the datasets generated and/or analysed during the current study are available from the corresponding author on reasonable request.
