# Supplementary material for: Oncologist approaches to communicating uncertain disease status in pediatric cancer: a qualitative study
Source: BMC Cancer. 2022 Oct 31;22:1109. doi: 10.1186/s12885-022-10190-6 (PMC9620648; doi:10.1186/s12885-022-10190-6)
Supplement: Supplementary file 1 — Supplementary Material 1 [file 12885_2022_10190_MOESM1_ESM.docx]

**Supplemental Table 1. CORE-Q (COnsolidated criteria for REporting Qualitative research) Checklist**

| Item No. | Topic | Guide Questions/Description | Reported on Page No. |
| --- | --- | --- | --- |
| **Domain 1: Research team and reflexivity** | | | |
| Personal characteristics | | | |
| 1 | Interviewer/facilitator | Which author/s conducted the interview or focus group? We provide the initials of the researchers on the study team who conducted interviews. | Methods, paragraph 3 |
| 2 | Credentials | What were the researcher’s credentials? We describe the credentials of the researchers on the study team. | Supplemental Table 2 |
| 3 | Occupation | What was their occupation at the time of the study? We describe the occupations of the researchers on the study team. | Supplemental Table 2 |
| 4 | Gender | Was the researcher male or female? We describe the genders and provide initials of the researchers on the study team. | Supplemental Table 2 |
| 5 | Experience and training | What experience or training did the researcher have? We describe the experience and training of the researchers on the study team. | Supplemental Table 2 |
| Relationship with participants | | | |
| 6 | Relationship established | Was a relationship established prior to study commencement? The researchers did not have a relationship with parents prior to the study. The researchers did know the oncologist participants in a professional capacity prior to the study. | Table 1 |
| 7 | Participant knowledge of the interviewer | What did the participants know about the researcher? (e.g., personal goals, reasons for doing the research) Participants knew that the researchers aimed to conduct this study to better understand how oncologists communicate about disease reevaluation findings with patients with cancer and their families. | Table 1 |
| 8 | Interviewer characteristics | What characteristics were reported about the interviewer/facilitator? e.g., bias, assumptions, reasons and interest in the research topic? Researchers who conducted interviews included a physician-scientist with training in communication, palliative care, and pediatric oncology and a Clinical Research Associate (CRA) with training in qualitative research. The research team was interested in this topic because of professional experiences that suggested that opportunities existed to improve communication about prognosis. | Supplemental Table 2 |
| **Domain 2: Study design** | | | |
| Theoretical framework | | | |
| 9 | Methodological orientation and theory | What methodological orientation was stated to underpin the study? e.g., grounded theory, discourse analysis, ethnography, phenomenology, content analysis. Content analysis was used in this study. | Methods, paragraph 6 |
| Participant selection | | | |
| 10 | Sampling | How were participants selected? e.g., purposive, convenience, consecutive, snowball. We used a convenience sample of oncologists who practice in the Solid Tumor clinic at a pediatric cancer center and their patients/families. | Table 1 |
| 11 | Method of approach | How were participants approached? e.g., face-to-face, telephone, mail, email. We approached participants to gauge interest either face-to-face, by telephone, or by email, and we completed formal consents in face-to-face discussions. | Table 1 |
| 12 | Sample size | How many participants were in the study? This study presents data for the 17 patient-parent dyads that experienced at least one disease reevaluation discussion about equivocal data while on study, representing all participating oncologists (n=6). | Table 2 |
| 13 | Non-participation | How many people refused to participate or dropped out? Reasons? As published previously, out of 41 patient-parent dyads approached, 7 individuals (patient or parent) declined to participate due to hesitation or refusal by the patient (n=3), parent (n=3), or both (n=1). No participants dropped out of the study. | Results, paragraph 1 |
| Setting | | | |
| 14 | Setting of data collection | Where was the data collected? e.g., home, clinic, workplace. Medical conversations were recorded in the clinic or hospital setting, as well as rarely via telephone if patients/families were unable to come to the hospital to learn their disease reevaluation findings. | Methods, paragraph 2 |
| 15 | Presence of non-participants | Was anyone else present besides the participants and researchers? Other clinicians and family or friends of the patient were present at times during recorded discussions. | Table 1 |
| 16 | Description of sample | What are the important characteristics of the sample? e.g., demographic data, date. Demographic data for participants are presented in Table 2; dates of medical discussions are not presented as these might be identifiers and are not relevant for interpretation of findings. | Table 2 |
| Data collection | | | |
| 17 | Interview guide | Were questions, prompts, guides provided by the authors? Was it pilot tested? Interview prompts were read aloud to participants. The interview guide was pilot tested with bereaved parents and oncologists prior to use. | Methods, paragraph 3 |
| 18 | Repeat interviews | Were repeat interviews carried out? If yes, how many? Yes, some participants completed repeat interviews because interviews were conducted following each recorded disease reevaluation conversation in which disease progression was discussed. | Methods, paragraph 3 |
| 19 | Audio/visual recording | Did the research use audio or visual recording to collect the data? Disease reevaluation discussions were audio-recorded in real time. | Methods, paragraph 3 |
| 20 | Field notes | Were field notes made during and/or after the interview or focus group? Researchers wrote memos following interviews. | Methods, paragraph 3 |
| 21 | Duration | What was the duration of the interview or focus group? Interviews ranged from 5 minutes to more than 2 hours in length, depending on the preference of the participant at times. Most interviews were approximately 20 minutes. | Methods, paragraph 3 |
| 22 | Data saturation | Was data saturation discussed? We recorded all disease reevaluation discussions for participating dyads until death or 24 months since disease progression on study. In this context, saturation was not relevant in terms of stopping data collection. However, we describe how saturation was achieved with respect to coding. | Methods, paragraph 5 |
| 23 | Transcripts returned | Were transcripts returned to participants for comment and/or correction? We did not consent patients/parents to return transcripts or data synthesis, as this was not felt to be appropriate in the context of recorded medical dialogue (as opposed to interviews), particularly given that most children had died and parents were bereaved at study end. | n/a |
| Data analysis | | | |
| 24 | Number of data coders | How many data coders coded the data? We provide initials to identify each research team member who coded data. | Methods, paragraph 6 |
| 25 | Description of the coding tree | Did authors provide a description of the coding tree or codebook? We present the full codebook in Supplemental Table 2. | Supplemental Table 3 |
| 26 | Derivation of themes | Were themes identified in advance or derived from the data? Themes were inductively derived from raw data. | Methods, paragraph 7 |
| 27 | Software | What software, if applicable, was used to manage data? We used MAXQDA software. | Methods, paragraph 6 |
| 28 | Participant checking | Did participants provide feedback on the findings? We did not consent patients/parents to provide feedback on findings, as this was not felt to be appropriate, given that most children had died and parents were bereaved at study end. | n/a |
| Reporting | | | |
| 29 | Quotations presented | Were participant quotations presented to illustrate the themes/findings? Was each quotation identified? e.g., participant number. Representative quotes are embedded within the text, with additional quotes presented in the comprehensive Tables 4 and 5. | Table 4; Table 5 |
| 30 | Data and findings consistent | Was there consistency between the data presented and the findings? We demonstrate consistency between data presented in the Results section and interpretation of findings delineated in the Discussion section. | Results/Discussion sections  Table 5; Table 6 |
| 31 | Clarity of major themes | Were major themes clearly presented in the findings? We presented all major themes in detail. | Table 5 |
| 32 | Clarity of minor themes | Is there a description of diverse cases or discussion of minor themes? We provide a variety of rich quotes in the text and in a comprehensive table to offer readers diversity of cases. | Table 4;  Table 5 |

Developed from: Tong A, Sainsbury P, Craig J. Consolidated criteria for reporting qualitative research (COREQ): a 32-item checklist for interviews and focus groups. *International Journal for Quality in Health Care*. 2007. Volume 19, Number 6: pp. 349 – 357.

**Supplemental Table 2. Research Team Attributes and Qualifications**

| A.P. | Female, white physician-scientist with a Medical Degree, a Ph.D. in Anthropology with a focus on medical anthropology, research experience in qualitative clinical research, and clinical training and practice in pediatrics and pediatric complex care. |
| --- | --- |
| C.W. | Female, African American clinical research associate with formal MAXQDA training and expertise in qualitative research methodology. |
| M.S. | Female, white physician-scientist trainee with a Medical Degree, training in pediatrics, and training in MAXQDA and qualitative research; currently completing pediatric hematology-oncology fellowship with a research focus in communication among clinicians, patients with cancer, and their families. |
| S.V. | Female, Southeast Asian-American graduate student (MPH) with training in MAXQDA and content analysis. |
| J.B. | Male, white physician-scientist with a Medical Degree, extensive clinical and research expertise related to difficult communication in oncology, and clinical training and practice in pediatric hematology-oncology and hospice and palliative medicine. |
| J.M. | Female, white physician-scientist with a Medical Degree, a Master’s in Public Health, extensive research expertise in communication science, clinical training in pediatric hematology-oncology and hospice and palliative medicine, and practice in pediatric hematology-oncology. |
| E.K. | Female, white physician-scientist with a Medical Degree, a Master’s in Public Health, graduate-level training in qualitative research methodology with a focus on communication science, and clinical training and practice in pediatric hematology-oncology and hospice and palliative medicine. |

**Supplemental Table 3. Prognostic Communication in Pediatric Cancer Codebook**

| **Code Title** | **Code Definition and Examples** |
| --- | --- |
| **Assessing prognostic understanding** | **Assessing the patient’s understanding of his/her prognosis.**  Includes understanding about future quality of life, functioning, ability to go to an event, general survival, etc.)  e.g., What do you understand about your prognosis?  What have your doctors told you about what’s likely to happen in the  future?  Are you worried about being able to attend your son’s graduation in a  few months from now? |
| **Prognostic uncertainty** | **Any mention of uncertainty with regard to estimating prognosis, guessing if a treatment will work, etc.**  Includes any mention of “I don’t know,” “not sure,” “unclear,” “hard to say,” etc. [Note: this code may dovetail with other prognostic communication codes, depending on the language used.]  e.g., I hope that this treatment will work, but I don’t know if it will.  It is difficult for us to know what the future holds.  We aren’t sure if your child will survive. |
| **Disease changing for the worse** | **Discussion of how the disease trajectory is changing for the worse without explicit mention of survival time or curability.**  Includes discussion of tumor sizes increasing, cancer spreading/ progressing, cancer unresponsiveness to treatment, or stating that things are getting worse. [Note: also apply subcodes for Pop_Ind and Clarity.]  e.g., The fact that the disease has increased in size tells me that we need to  change course.  Now that disease is in your bones, I think it’s likely that it has spread.  elsewhere in your body.  The lesions in your lungs are getting bigger. |
| **Best and worst-case scenarios** | **Discussing best case and worse care scenarios.**  Be sure there is a clear top and bottom anchor. If there is only a top OR bottom anchor, code as survival time. [Note: also apply subcodes for Pop_Ind and Clarity.]  e.g., Most patients will survive 6- 12 months. Some may do better and live  up to 2 years, and some do worse- only 2-3 months. |
| **Survival time** | **Discussing estimates of survival time.**  [Note: also apply subcodes for Pop_Ind and Clarity.]  e.g., I’m not sure that you will make it to Christmas this year.  I think we’re talking weeks to months, instead of months to years.  I think there is at least a small chance that you will have long term  control after radiation.  It’s important that you start doing the things on your bucket list. If  there is something that you would like to make sure you have a chance  to do, whatever it is, then you should try to do it. |
| **Curability**  ***Subcodes***  **Curability**  **Clarity**  **Population vs. Individual** | **Discussing if the cancer can be cured.** “Curability” refers to the ability or potential to cure a disease, irrespective of prognosis (favorable or poor). Any language conveying information about a patient’s chance for survival could be coded as “curability;” inclusion of the words “cure” or “prognosis” was not required. Includes discussion of cancer not going away or not being treatable. [Note: also apply subcodes for Clarity and Pop_Ind.]  e.g., Although the treatments might help you live longer and live better,  they cannot completely get rid of the cancer. It’s not a cure.  **No Cure:** Not curable.  e.g., Unfortunately, we can’t get rid of your illness.  I wish that we could make your cancer go away, but sadly we cannot.  **Unlikely Cure:** Cure is not likely to be achieved.  e.g., Our goal is to try to shrink the tumor, but I doubt that the  chemotherapy and the radiation will make it go away.  **Likely Cure:** Cure is likely to be achieved.  e.g., Your body is responding well to the chemotherapy. It is shrinking the  tumors and it looks like we might be able to get rid of all the cancer  cells.  **Clear (direct):** When a curability statement uses direct/frank language to express how disease progression will affect the patient’s future life.  e.g., You’ve got an illness where you, you know, this one we can’t get rid  of. We can’t cure your cancer.  **Cloudy (indirect):** When a prognostic statement implies a poor outcome, but does not directly state it (i.e., one may read between the lines to assume how disease progression will affect the patient’s future life)  e.g., It’s different for every person. But the fear is that sometime down the  line we’re gonna see cancer elsewhere in your body.  There is some evidence that things are starting to progress.  **Population:** Speaking generally to all patients with this cancer type.  e.g., So, generally stage 4 cancer is not curable because the tumor always  tends to come back.  **Individual:** Speaking to the specific patient or family.  e.g., And we expect eventually, whether six or twelve months, your disease  should progress. |

**Additional Files Information**

Supplemental Table 1. CORE-Q (COnsolidated criteria for REporting Qualitative research) Checklist, Microsoft Word document (.docx)

Supplemental Table 2. Research Team Attributes and Qualifications, Microsoft Word document (.docx)

Supplemental Table 3. Prognostic Communication in Pediatric Cancer Codebook, Microsoft Word document (.docx)
